# The Anthro-Thumb: a biomimetic hybrid soft robotic carpometacarpal saddle joint for the thumb

**DOI:** 10.3389/frobt.2025.1496073

**Published:** 2025-04-29

**Authors:** Declan Symes, Michael Rose, Emanuel David Nunez Sardinha, Aghil Jafari, Javeed Hussain, Appolinaire Etoundi

**Affiliations:** ^1^ School of Engineering, University of the West of England (UWE), Bristol, United Kingdom; ^2^ Department of Electrical and Electronic Engineering, Global College of Engineering and Technology, Muscat, Oman

**Keywords:** Biomimetic soft robotics, carpometacarpal saddle joint, robotic thumb design, thumb biomechanics, dexterity, soft robotics, anthropomorphic

## Abstract

Robotic hand design is multifaceted, with the design of robotic thumbs often oversimplified to facilitate ease of manufacture, control, and reliability. Despite the extensive development of robotic hands, the need for a more dexterous and anthropomorphic thumb design remains significant, particularly for applications in prosthetics and rehabilitation robotics, where naturalistic movement and adaptability are essential. This paper addresses this gap by exploring the conception, evolution, and evaluation of a unique biomimetic soft thumb. The thumb plays a vital role in hand function, and its unique range of motion is enabled by the carpometacarpal (CMC) saddle joint. By harnessing the biologically accurate mechanisms of the CMC joint, this research aims to enhance the functionality of tendon-driven robotic hands, offering improved dexterity and adaptability for tasks such as grasping and manipulation. The introduced *Anthro-Thumb* is a biomimetic soft robotic thumb that provides a comprehensive range of motion at the thumb’s base while ensuring cost efficiency and reduced mechanical complexity. A comparative analysis with existing robotic thumb designs highlights the advancements of the *Anthro-Thumb*, particularly in terms of range of motion and cost-effectiveness. Additionally, we discuss the long-term durability and maintenance requirements of the soft robotic materials and components used. When subjected to the Kapandji physiotherapy test, the design secured a commendable score of 9 of 10, with 10 representing the highest level of dexterity achievable by a human thumb. The findings affirm that employing biomimetic soft-structured robotic CMC saddle joints is a promising strategy to address the challenges associated with robotic thumb development in robotic hands.

## 1 Introduction

The thumb plays a vital role in hand function, and without it acting as an opposing digit, most of the hand’s capabilities are lost (Beyer, 2016). This is because the thumb’s opposition movement is critical for enabling fine motor skills such as grasping, pinching, and manipulating objects. The carpometacarpal (CMC) joint, a type of saddle joint, enables the thumb’s unique range of motion (ROM), including abduction, adduction, flexion, extension, and circumduction. The design presented demonstrates the use of a biomimetic saddle joint located at the base of the thumb to provide the two required degrees of freedom (DOFs) to the first metacarpal. Without this functionality, the hand’s ability to interact effectively with tools and objects would be significantly impaired. Although this has been acknowledged for decades, the development of thumb systems for robotic hands has been sparse (Chalon et al., 2010). Many robotic hands have been developed to replicate the functions achievable by human hands, yet these often sacrifice the unique functionality of the thumb to enhance reliability and simplify design (Mohammadi et al., 2020) or incorporate very complex driving systems that significantly increase costs, limiting wider adoption (Lee et al., 2017; Chapman et al., 2022; Butterfass et al., 2004). However, some robotic hands have been designed to be anatomically correct, including the CMC joint, such as those discussed in *Mechanisms of the Anatomically Correct Testbed Hand* (Deshpande et al., 2011) and *Design of the Anatomically Correct*, *Biomechatronic Hand* (Tasi et al., 2019).

Following current trends in robotics, this paper aims to develop an anthropomorphic robotic thumb to achieve dexterity comparable to human hands and other robotic systems at a low cost using primarily common 3D-printed structures and soft silicone elements. Increasing the anthropomorphic aspects of an engineered system is assumed to improve its capabilities. Thus, a biomimetic approach is adopted to develop a soft robotic thumb that emulates the functionality of a biological thumb by imitating joint positions and incorporating biologically beneficial features.

**FIGURE 1:**
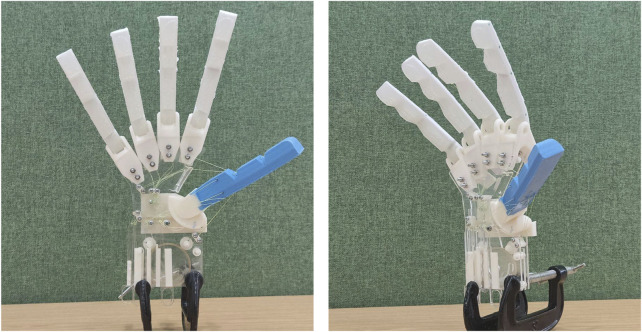
Final hand featuring the 3D-printed, biomimicking CMC saddle joint embedded into the soft robotic *Anthro-Thumb*.

The design presented, shown in Figure 1, demonstrates the use of a biomimetic saddle joint located at the base of the thumb to provide the two required DOFs to the first metacarpal. This biomimetic system enables anthropomorphic movement of the thumb, improving the realism of the hand and aligning its dexterity with that of a biological hand. Embedding the joint into a soft robotic thumb retains the benefits of using a singular soft material structure (Sardinha et al., 2022) while incorporating the full ROM aspired for in a biomimetic thumb. The CMC joint of the thumb enables abduction, adduction, flexion, (passive) extension, and circumduction via a series of four controlling pseudo-muscles (cables mapped to the origin and the attachment of each muscle of the thenar eminence and the adductor pollicis).

The design methodology for this work is described in the following sections: in Section 2, we provide a brief overview of current robotic thumbs and thumb anatomy, and we determine the requirements of the robotic thumb. In Section 3, we design the biomimetic soft robotic hand, comprising the CMC saddle joint, pseudo-capsular ligament, pseudo-muscles, first metacarpal, and soft robotic exterior. We build upon the fingers developed by Sardinha et al. (2022), which provide easy-to-control human-like synergies and compliance in a simple and low-cost form factor. We also detail the fabrication process of the thumb itself. Section 4 describes the results and testing methodology for the resulting design, along with a brief validation. In Section 5, we provide a technical discussion of the success of the development of the *Anthro-Thumb*, and we provide conclusions in Section 6.

## 2 Related works

This section provides a brief overview of current robotic hands, enabling the identification of different joint types and ROMs demonstrated in the field of robotic thumbs. It also highlights areas where further development can take place, culminating in the derivation of design requirements.

The saddle joint in the human thumb provides two DOFs at the base of the thumb. The robotic hands discussed below demonstrate that although efforts have been made to develop anthropomorphic designs, the thumb’s design is often compromised to prioritize manufacturing, maintainability, or simplicity. As a result, robotic thumbs lack human-like functionality, which limits the anthropomorphism of the entire hand. When the thumb is designed to accurately replicate human functionality, the complexity of the design becomes significantly high (Butterfass et al., 2004).

This increased complexity often leads to the use of two independent hinge joints to mimic the thumb’s functionality (Chapman et al., 2022; Kontoudis et al., 2019; Zhou et al., 2018; Lee et al., 2017). Other robotic hand designs opt to freeze the first metacarpal in place, actuating only the two phalanges of the thumb, as seen in the KIT prosthetic hand (Weiner et al., 2018). Alternatively, some designs utilize entirely soft inflatable structures (Deimel and Brock, 2015) or implement more accurate three-DOF systems with underactuated elements (Chapman et al., 2022).

Although these methods provide varying levels of functionality, they often result in deviations from the natural appearance of a hand, unnatural object grasping positions, or reduced dexterity. Developing an accessible saddle joint for a robotic hand would enable a more natural grasp and introduce the two DOFs observed in a biological hand.

Weiner et al. (2018) discuss the design and control of the KIT prosthetic hand, a novel five-finger 3D-printed prosthesis. The KIT hand utilizes two motors to achieve a total of 10 DOFs for the hand via a series of internally routed tendons. The 3D-printed hand features an anthropomorphic palm that serves as a chassis for the multi-part robotic fingers while enabling a natural gripping motion. The thumb consists of two phalanges (proximal and distal), with the 3D-printed palm including the first metacarpal in a fixed flexed position. This positions the thumb appropriately for a standard gripping motion. However, it is worth noting that freezing the first metacarpal limits the hand’s functionality to the reduced ROM provided by the single DOF on each of the thumb’s two phalanges.

Chalon et al. (2010) focus on providing guidelines for designing robotic thumbs and consider that although the vital role of the thumb in hand performance has been acknowledged for decades, it has been sparsely addressed by roboticists. They provide guidance on the anatomy and ROM of joints and establish links between robotic hand design, surgery, and anatomy.

The Kapandji test, in particular, is recommended as a method of measuring the ROM and opposition that can be achieved by a designed thumb.

Table 1 presents a compiled set of self-reported Kapandji test scores for popular high-end robotic hands. The scale on which the test results are reported varies slightly, partly due to differences in test protocols and partly because some designs show minimal distances between successive points of contact, particularly at the metacarpophalangeal (MCP) joint and palmar crease.

**TABLE 1:** Self-reported Kapandji test scores for other popular dexterous robotic hands and thumb actuation strategies.

Hand	Kapandji test result	Thumb joint strategy
S-22 (Zhou et al., 2020)	10	Three fully actuated hinge joints
Chapman et al. (2022)	9 of 11	Three DOFs, one underactuated. One motor for flexion/extension via soft tendons. Two coupled DOFs for thumb opposition and abduction/adduction
DLR Hand II (Grebenstein et al., 2010; Butterfass et al., 2004)	11 of 11	Three DOFs, fully actuated. Saddle joint and hinge joints
Kontoudis et al. (2019)	“Completed” (8 shown)	Two actuated DOFs: flexion/extension and bidirectional abduction/adduction
BCL-13 (Zhou et al., 2018)	8 of 10	Two fully actuated hinge joints
KITECH-Hand (Lee et al., 2017)	10 of 10	Two fully actuated hinge joints
RBO Hand 2 (Deimel and Brock, 2015)	7 of 8	Two pneumatic actuators
RBO Hand 3 (Puhlmann et al., 2022)	“Highest possible” (10 shown)	Three actuated DOFs for flexion/extension, abduction/adduction, and anteposition/reposition as hinge joints

All the designs with a self-reported maximum or perfect Kapandji score use fully actuated hinge joints in series for the thumb MCP, driven by traditional “rigid” control mechanisms, either in two DOFs (Kontoudis et al., 2019; Lee et al., 2017) or three DOFs (Zhou et al., 2020; Butterfass et al., 2004; Puhlmann et al., 2022). Of these, only the DLR Hand (Butterfass et al., 2004) explicitly targets a saddle joint to achieve parity with human joints, resulting in a successful but complex design. Although recent works by Shorthose et al. (2022) have demonstrated soft robotic hands with impressive dexterity, our design uniquely integrates a biomimetic CMC saddle joint with soft materials, achieving a high level of anthropomorphic accuracy and functionality.

The RBO Hand 2, a purely soft inflatable hand, achieves all but the last point in the MCP joint (Deimel and Brock, 2015). The approach by Chapman et al. (2022), which combines traditional hard robotics with an underactuated soft joint, misses the last two touchpoints, where the little finger MCP and the equivalent of the palmar crease could not be reached.

To develop a robotic thumb that utilizes a true saddle joint to provide the full, independent ROM and DOF expected of an anthropomorphic thumb, a thorough understanding of the anatomy of the human thumb is required.

### 2.1 Thumb anatomy

To enable the development of a soft robotic thumb with improved anthropomorphic accuracy, it is essential to understand the structure of the human thumb, its functionality, and how it differs from the other fingers of the hand. In this section, the various elements of thumb anatomy are explored from a mechanical perspective, starting with the structure (bones), followed by methods of actuation (muscles), and finally the mechanics of movement (joints and ROM). A series of technical requirements for designing an anthropomorphically accurate thumb is then derived. Using these requirements, areas where the biological design can be mimicked are identified and incorporated into the subsequent design stage.

The osteoarticular structure of the thumb system consists of five bones: the scaphoid carpal bone, the trapezium carpal bone, the first metacarpal, the proximal phalanx, and the distal phalanx, which are arranged from the most proximal to the most distal (Beyer, 2016). These bones interface through a series of joints: the CMC joint between the trapezium and the first metacarpal, the MCP joint between the first metacarpal and the proximal phalanx, and the interphalangeal (IP) joint between the proximal and distal phalanges (Henry Gray and Carter, 2010). The MCP and IP joints behave similarly to the other finger joints in the hand, presenting hinge-like mechanisms, but with one less phalanx in the thumb. The CMC joint, however, functions as a saddle joint, providing an extended range of movement compared to the other digits of the hand. A capsular ligament surrounds the CMC saddle joint, connecting the first metacarpal to the trapezium carpal bone, thereby providing stability and strength (Henry Gray and Carter, 2010).

The saddle joint relies on two interfacing concave–convex surfaces to facilitate flexion–extension, abduction–adduction, and circumduction while prohibiting axial rotation (Henry Gray and Carter, 2010). *Abduction* moves the thumb outward, away from the rest of the hand, whereas its counterpart, *adduction*, pulls the thumb inward toward the index finger. *Flexion* moves the thumb over the palm toward the little finger, and *extension* relaxes the thumb away from the little finger. *Circumduction* is the circular movement of the metacarpal, forming a conical shape. These movements allow the thumb to oppose the fingers of the hand, enabling grasping mechanisms. The arches of the hand, particularly the longitudinal and transverse arches, create a concave surface in the palm that supports the thumb’s ROM. This concavity is essential for enabling opposition and circumduction of the thumb. In the *Anthro-Thumb* design, the robotic metacarpal bones were planned with a concave structure to mimic this anatomical feature, ensuring a more natural and functional ROM.

These movements are produced by the thenar muscle group (the flexor pollicis brevis, the opponens pollicis, and the abductor pollicis brevis) on the palmar side of the hand, as well as by the adductor pollicis (Henry Gray and Carter, 2010).

1. The flexor pollicis brevis connects to the capitate carpal bone and the outside of the proximal phalanx via a thin tendon, flexing the thumb toward the small finger.
2. The opponens pollicis connects to the trapezium and annular ligament of the wrist, as well as the whole length of the radial side of the first metacarpal (Henry Gray and Carter, 2010), rotating the thumb so that it opposes the tips of the other fingers.
3. The abductor pollicis brevis connects to the trapezium and the base of the proximal phalanx via a tendon, abducting the thumb by pulling it away from the index finger.
4. The adductor pollicis connects both the second and third metacarpals to the inner side of the proximal phalanx, adducting the thumb by pulling it closer to the index finger.

The ROM indicates how far each joint can move in either direction. It has been documented to change throughout life, influenced by factors such as age, health, and gender, and the overall mean CMC ROM values for humans have been provided (White et al., 2018).

The paper focusing on guidance for robotic thumb design (Chalon et al., 2010) offers comprehensive CMC ROM values and suggests a central resting position for the robotic thumb.

Table 2 presents the suggested ROM for both the mean biological hand CMC and the guidance for robotic thumb design. As the second study is specifically oriented toward robotic thumb development and provides more detailed insights into the resting position of the first metacarpal, it has been incorporated into the requirements set for a thumb design.

**TABLE 2:** CMC joint range of movement (ROM).

Test	Adduction/abduction (deg)	Flexion/extension (deg)
Mean biological hand CMC ROM (White et al., 2018)	51.1°	21.7°/19.5°
Robotic thumb guidance CMC ROM (Chalon et al., 2010)	35°/25°	15°/30°
Robotic thumb guidance CMC resting position (Chalon et al., 2010)	35° from the second metacarpal	30° from the second metacarpal

### 2.2 Requirement derivation

Based on the above information and additional ergonomic hand data from an anthropometric study of a male hand conducted by the U.S. Army (Gordon et al., 1989), it is possible to derive requirements for an anthropomorphically accurate biomimetic soft robotic thumb. Requirements were determined based on size, anthropomorphic accuracy, ease of control, and biomimicry to facilitate thumb functionality. The requirements identified below have been developed to enable the design of the soft robotic thumb. Each requirement includes at least one level of compliance: threshold (the minimal acceptable level of performance) and, where applicable, an objective stretch target.

Through an assessment of the thumb against the requirements, four areas were identified where biological design can be utilized and mimicked in the development of this robotic thumb. The thumb MCP and IP joint wedge angles were designed to allow approximately 50° and 80° of flexion, respectively, based on the anatomical data. For the other fingers, separate wedge angles were planned for the DIP and PIP joints to reflect the natural ROM of each digit. These angles were incorporated into the silicone molds to ensure realistic finger movement. First, the joint at the base of the robotic thumb has been developed to mimic that of a biological thumb. Second, a system has been designed to replicate the function of the capsular ligaments. Third, a rigid first metacarpal has been developed to provide structure to the soft robotic thumb, representing the skeletal framework of the thumb. Finally, the control mechanisms used to manipulate the thumb take inspiration from the controlling muscles of a biological thumb. The requirements for the Anthro-Thumb were derived from the anatomical features of the human thumb, as detailed in Section 2.2. These requirements align with the four areas of biomimicry identified earlier: the CMC joint, capsular ligaments, first metacarpal, and control mechanisms.

*Requirement 1*: *size—base*. Threshold: the overall size (width and length) should be within 10% of the 50% adult male per Loisel et al. (2015), in addition to a space claim for the CMC joint. Objective: same as the threshold but including the thickness of the base.

*Requirement 2*: *size—CMC joint*. Threshold: the CMC joint should be proportional in size to the overall hand. Objective: N/A.

*Requirement 3*: *size—metacarpal*. Threshold: the overall size (width and length) should be within 10% of the 50% adult male per Gordon et al. (1989), in addition to a space claim for connection to the phalanges. Objective: same as the threshold but including the circumference of the metacarpal.

*Requirement 4*: *control*. Threshold: the movement of the metacarpal should utilize anthropomorphically accurate force locations (i.e., cables/muscles shall connect in biologically accurate locations). Objective: N/A.

*Requirement 5*: *biomimicry—CMC joint*. Threshold: the CMC joint should provide the same ROM as a natural CMC joint. Objective: same as the threshold but additionally functions in the same way as a natural CMC joint.

*Requirement 6*: *connecting fixtures*. Threshold: the base should have a space claim and fittings to enable the connection of the base to the CMC joint. The CMC joint should have fittings to enable connection to the base and the metacarpal. The metacarpal should have fittings to enable connection to the CMC joint and phalanges. Objective: N/A.

*Requirement 7*: *control methods*. Threshold: the system should incorporate a biologically inspired method for controlling the thumb to achieve the desired CMC joint movement. Objective: N/A.

## 3 Materials and methods

This section covers the design process for the *Anthro-Thumb*, including the design of the CMC joint and its ligamentous and muscle structure. The design of the hand frame is then discussed, followed by the construction of the fingers, based on the methodology developed for the soft hand presented in Sardinha et al. (2022). Finally, the final assembly and its integration are presented.

### 3.1 Thumb base

The design of the *Anthro-Thumb* consists of two 3D-printed structures replicating the behavior of the first metacarpal bone mounted onto the trapezium carpal bone by a saddle joint (CMC). It was 3D-printed using PLA, a rigid material, whereas the surrounding structure and fingers were fabricated from soft silicone to achieve compliance and adaptability. The structure supports and enables the actuation of an embedded soft robotic thumb, stabilized using a pseudo-capsular ligament and controlled by a pseudo-muscle. The distal joints are entirely soft, allowing the thumb to deform and adapt to contact surfaces during grasping.

The saddle joint was designed by applying the ROM dimensions. The values for the inner arcs of the concave–convex surfaces were adapted from the 50% of a male thumb dimensions (Gordon et al., 1989) and rounded to the nearest tenth of a millimeter for simplicity. For the first metacarpal, the convex surface uses a radius of 20 mm, and the concave surface uses 21 mm to allow for smooth movement within the joint.

The trapezium carpal bone section of the CMC joint is angled to position the thumb in a natural resting orientation, reflecting the alignment of the first metacarpal relative to the second metacarpal. A flat plate is extruded from the base of the concave–convex surface to allow mounting onto the dorsal plate of the robotic hand frame.

**FIGURE 2:**
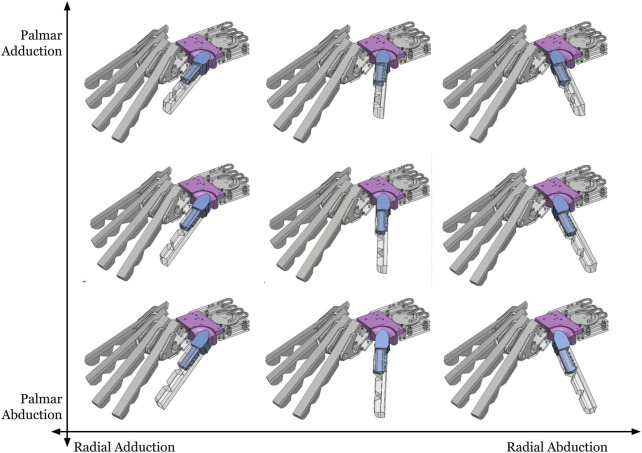
Thumb ROM for the saddle joint. Movements are the closest analogs to abduction/adduction of the palm (vertical axis) and radius (horizontal axis).

The ROM values (abduction, adduction, flexion, and extension) were mapped onto these surfaces to determine the points where the concave surface would impact the rotation of the convex surface. This process identified areas requiring modification to restrict movement and achieve the desired ROM. Physical stops were introduced to both sides of the saddle joint to prevent over-extension, and stability issues identified in early prototypes were addressed. A ridge was added to the top of the trapezium carpal bone to prevent over-flexion of the joint, and stopper blocks were added to the radial and ulnar sides of the thumb to prevent over-abduction and over-adduction, respectively. Figure 2 shows the end-point saddle joint positions for different actuation values.

**FIGURE 3:**
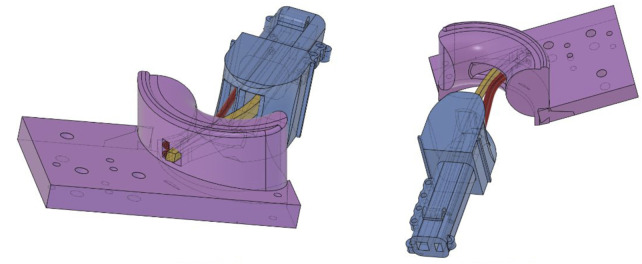
Detailed view of the thumb canal (palmar side), showing the elastic tendon (yellow) and two tendon channels (red).

The articular surfaces of the biological CMC joint are connected by a capsular ligament (Henry Gray and Carter, 2010), which provides strength and stability, keeping the first metacarpal in place (Henry Gray and Carter, 2010). This is crucial for the function of the joint, as there is no physical connection between the concave–convex surfaces of the trapezium and the first metacarpal. To mimic the function of a biological capsular ligament, an internal “ligament” was devised to provide dispersed stability to the joint. This “ligament” runs centrally within the two concave–convex surfaces and is securely anchored at both ends. The ROM required for both adduction/abduction and flexion/extension was incorporated into the design of the internal canal through which the mimicking ligament runs, as shown in Figure 3. The saddle joint was designed by applying the ROM dimensions derived from the 50th percentile male thumb.

External elastic harnesses that better resemble the biological capsular ligament were considered but dismissed, as they would be exposed and less protected. The internal ligament approach was, therefore, incorporated into the system.

The control of the CMC joint is primarily achieved through the use of the four muscles described in Section 2.1. A tendon is used to drive the joint, following the locations of the thenar muscles and the adductor pollicis, enabling the control of the first metacarpal. The forces are applied in the same direction as the muscles would act to control the biological CMC joint.

The first metacarpal forms one half of the saddle joint and acts as a structural internal component of the soft robotic thumb. It is a narrow structure with fixing points for all pseudo-muscles to ensure strong attachment. This metacarpal features an internal channel that holds both the interior ligament and the driving tendon, which controls the flexion (proximal and distal phalanges) of the thumb while maintaining its position. This channel is aligned with the channel in the trapezium carpal bone (the second half of the saddle joint) and uses silicone tubes to hold the tendon securely.

Although not all of the muscles in a biological thumb attach to the first metacarpal, it was determined that due to the absence of a rigid proximal phalanx in the soft robotic thumb, attaching the pseudo-muscles at the tip of the first metacarpal instead of the base of the phalanx was suitable. Each pseudo-muscle was inserted at the following locations on the thumb:

1. **Abductor pollicis brevis:** this muscle is inserted at the base of the proximal phalanx in a biological thumb (Henry Gray and Carter, 2010). Due to the absence of a physical proximal phalanx in the soft silicone thumb design, the top of the radial side of the first metacarpal was identified as the most suitable location for a fixture. A single loop was developed here to allow cable tubing to be securely inserted.
2. **Flexor pollicis brevis:** this muscle is also inserted at the base of the proximal phalanx in a biological thumb (Henry Gray and Carter, 2010). For the soft robotic thumb, the top of the palmar side of the first metacarpal was identified as the most appropriate location for a fixture.
3. **Opponens pollicis:** this muscle is inserted along the entire length of the radial side of the first metacarpal in a biological thumb (Henry Gray and Carter, 2010). Fixtures were designed to be mounted along the entire radial side of the first metacarpal. Four loops were introduced here to allow two cable tubes to pass through in U shapes, enabling the cabling to connect along the entire radial side.
4. **Adductor pollicis:** this muscle is inserted at the ulnar side of the proximal phalanx in a biological thumb (Henry Gray and Carter, 2010). Due to the lack of a physical proximal phalanx in the soft silicone thumb design, the top of the ulnar side of the first metacarpal was determined to be the most suitable location for a fixture. A single loop was developed here to allow cable tubing to be securely inserted.

### 3.2 Soft fingers

The design for the fingers was adapted from the soft synergy design presented by Sardinha et al. (2022) and was based on Manti et al. (2015). The fingers are soft silicone actuators driven by a central tendon running along their core. These actuators bend along a crease when tension is applied to the tendon, resulting in a grasping action. When the tension is released, the fingers passively relax and return to their base shape. An inner silicone tube is used to guide the tendon cable and prevent shearing. The soft compliant fingers can deform around irregular objects, providing natural adaptation to surfaces and enhanced resiliency.

Each finger is programmed to move according to the grasping synergy corresponding to the linearized *first principal component* as identified by Santello et al. (1998), which was achieved by sequentially modifying the relative thickness between successive joints. The fingers are wired together to a single actuation point, enabling simultaneous movement. This greatly simplifies operation, reducing the required input DOFs from 3 per finger (12 in total when excluding the thumb) to just 1 for all fingers.

#### 3.2.1 Materials

The soft silicone four front fingers (index, middle, ring, and small fingers) were fabricated using ALCHEMIX RTV 260 (Young’s modulus: 0.952 MPa) for the bendable structure and SILASTIC RTV-4234 (Young’s modulus: 0.494 MPa) for the contact area. These materials were chosen for their ability to mimic the compliance and adaptability of human fingers. The 3D-printed components, made of PLA, provided structural support while maintaining a lightweight design. The stiffer material (ALCHEMIX RTV 260) provides a strong backbone for the finger, ensuring stability, whereas the softer material (SILASTIC RTV-4234) provides a deformable contact area that adapts to the grasped objects.

For the thumb, due to its increased thickness relative to the other fingers, Smooth-Sil 950 was selected as the sole material. Its Young’s modulus, measured at 0.840 MPa, offers an intermediate stiffness compared to that of the front fingers, enabling the thumb to return to its base shape while maintaining a soft contact area at the fingertips. This material also avoids the need for multi-stage casting.

#### 3.2.2 Fabrication process

The fingers were constructed following the methodology in the original publication. Molds were designed and 3D-printed in PLA. A 2-mm-wide soft silicone tube was used to guide the tendons, passing through all fixture points to form channels for the pseudo-muscles during assembly. The fabrication process involved mixing of the two-part soft silicone materials, pouring the mixture into the mold, degassing it in a vacuum chamber, and curing it for 24 h. After curing, the silicone tubes were snipped off at the base of the mold, and the fingers were removed. The tubes were coated with the PTFE lubricant, and a thin fishing line (0.3 mm ∅ ) was used as the driving tendon.

#### 3.2.3 Thumb fabrication

**FIGURE 4:**
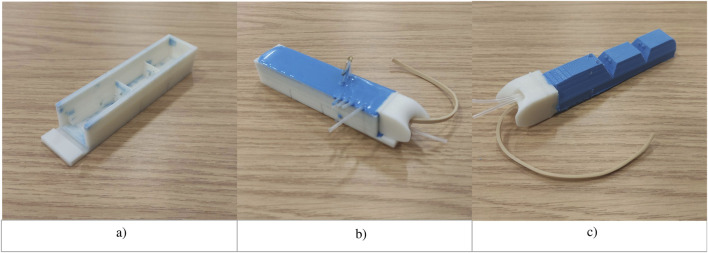
Fabrication process for the silicone soft robotic thumb. **(a)** Base mold. **(b)** Mold with metacarpal and silicone. **(c)** Silicone cured and thumb released.

For the *Anthro-Thumb*, a new internal structure—the first metacarpal (Figure 3)—was designed and 3D-printed in PLA. This metacarpal was inserted into a modified mold to form the shape of the soft thumb. The molding process involved mixing of Smooth-Sil 950 silicone, pouring it into the mold, degassing it in a vacuum chamber, and curing it for 24 h. After curing, the thumb with a bone-like internal structure (the first metacarpal) was demolded, demonstrating desired compliant characteristics and soft contact area. Figure 4 illustrates the fabrication process for the silicone soft robotic thumb.

### 3.3 Hand control mechanisms

To enable full control of the hand, five actions in the thumb can be actuated, corresponding to the muscles identified previously. This is complemented by the control of the fingers via synergies, as discussed, for a total of six individual actuation methods.

**FIGURE 5:**
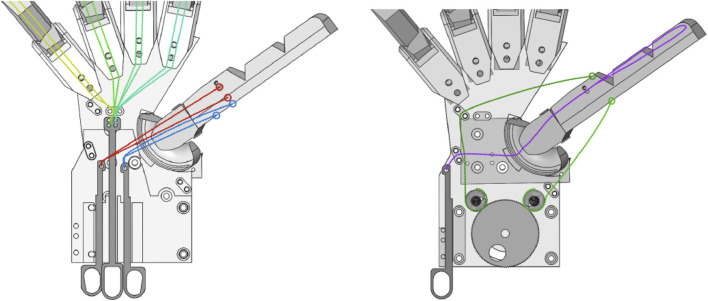
Hand controls on the different layers of the palm. Left image (from left to right): controls for front fingers (green and yellow), flexion of the thumb (red), and thumb opposition (blue). Right image (from left to right): controls for gripping of the thumb (purple) and wheel for abduction/adduction of the thumb.

These control mechanisms are distributed across two layers (palmar and dorsal) in the wrist of the hand to reduce the interference of cables crossing each other. Figure 5 shows the routing of each cable along with their respective control mechanisms. These movements are implemented through five independent controls, each emulating a different muscle or muscle group. All actions are passive, with the fingers returning to their starting position when no force is applied. The control system of the robotic hand is designed to mimic human dexterity while ensuring a smooth and coordinated movement. In Figure 5, the left image illustrates the routing of different control cables. The cables were made of nylon, which was chosen for its durability and flexibility. During testing, the hand was manually actuated using control pins, as motors were not yet integrated into the design. The green cables are responsible for actuating the front fingers, allowing them to flex and grasp objects in unison. The yellow cable, which serves the same function as the green cables, contributes to the coordinated movement of the fingers, ensuring that the hand maintains a natural grasping motion. The red cable controls the flexion of the thumb, enabling it to curl inward for a firm grip, whereas the blue cable manages the opposition of the thumb, allowing it to move across the palm for a more natural grasp. In the right image, the purple cable is responsible for gripping the thumb, adding to its versatility, whereas the green cables in this view are dedicated to the abduction and adduction of the thumb via a rotating wheel mechanism. The routing of all these cables has been carefully designed to prevent tangling and interference, ensuring that the hand operates efficiently and reliably while maintaining a high level of dexterity similar to that of a human hand.

#### 3.3.1 Palmar layer control mechanisms

The palmar layered flexible structure was designed to align with the natural concavity of the hand arches, ensuring that the Anthro-Thumb mimics the biomechanical properties of a biological hand. This design enhances the thumb’s ability to oppose the fingers and adapt to various grasping tasks.

1. **Actuation of the flexor pollicis brevis pseudo-muscle:** the cable representing the pseudo-flexor pollicis brevis enables flexion of the thumb toward the small finger. The cable is routed into the palm at the position of its biological origin (capitate carpal bone (Perkins and Hast, 1993)) and attached to the left handle of the palmar layer of control mechanisms. The handle length was determined based on the required distance to achieve full actuation of the pseudo-muscle.
2. **Actuation of the four fingers:** each of the four fingers is controlled by an individual cable running internally, as described in Sardinha et al. (2022). To enable simultaneous closing of all fingers, the cables are passed through a cable gate and attached to a single handle at the center of the palmar layer of control mechanisms. The handle length was determined based on the required distance for full actuation of the fingers.
3. **Actuation of the opponens pollicis pseudo-muscle:** the cable representing the pseudo-opponens pollicis enables the thumb to move into a position opposing the fingers. The cable is routed into the palm at the position of its biological origin (trapezium carpal bone and annular ligament of the wrist (Perkins and Hast, 1993)) and attached to the right handle of the palmar layer of control mechanisms. The handle length was determined based on the required distance to achieve full actuation of the pseudo-muscle.

#### 3.3.2 Dorsal layer control mechanisms

1. **Actuation of the proximal and distal phalanges of the thumb:** the actuation of the proximal and distal phalanges is controlled by the individual cable running internally in the thumb, similar to the mechanism used for the fingers (Sardinha et al., 2022). This cable is routed through the thumb, into the first metacarpal, through the CMC joint, and across the hand into a cable gate before being attached to a handle on the left of the dorsal layer of control mechanisms. The handle length was determined based on the required distance for full actuation of the proximal and distal phalanges.
2. **Abduction and adduction of the thumb:** although the actuation forces of the abductor pollicis brevis and adductor pollicis are individual functions, abduction and adduction of the thumb are antagonistic motions. These were implemented using a single bidirectional mechanism in the form of a wheel, combining the two motions into one control function. Each pseudo-muscle cable is routed through a cable gate and attached to a small gear at the center of the dorsal layer of control mechanisms. These gears are linked to a larger driving wheel that enables one pseudo-muscle to contract whereas the other relaxes.

The pseudo-abductor pollicis brevis is routed through a cable gate at its biological origin (radial edge of the trapezium (Perkins and Hast, 1993)) before being attached to its respective gear. The pseudo-adductor pollicis is routed into the curved section of the palmar surface before passing through a cable gate to avoid interference with other internal components and then attached to its respective gear.

### 3.4 Hand frame

The hand frame consists of three laser-cut acrylic plates that provide structural support for the various components of the hand. These plates—referred to as the palmar, middle, and dorsal plates—serve as mounting points for the thumb joint, finger-supporting structures, and control mechanisms.

The *palmar plate* holds the mounting points for the four fingers and acts as the palm of the hand. It includes all the channels required for the pseudo-muscles to pass through, mimicking the origin locations of the biological muscles in the hand. To replicate the natural curvature of the first crotch of the palm, the palmar plate was heated and bent to a 45° angle.

The *middle plate* serves as the mounting base for the control mechanisms of the pseudo-muscles in the hand. It ensures alignment and stability for the actuators that drive the fingers and thumb.

The *dorsal plate* holds the mounting point for the CMC joint of the thumb and functions as the back of the robotic hand. It provides stability and serves as the anchor for thumb-related components.

#### 3.4.1 Fabrication

The three hand plates, along with the handles for the control mechanisms, were laser-cut from 3-mm acrylic sheets. The physical saddle joint and finger mounts were 3D-printed and secured to the plates using screws.

## 4 Results

This section explores the results of the assessment of the Anthro-Thumb, including a discussion of its capabilities. Possible grasps are demonstrated using a small collection of items. The effective ROM is analyzed, and the minimum actuation force is measured. Thumb dexterity is evaluated using the Kapandji test.

The completed hand design is shown in Figure 1, displaying the threaded thumb and fingers, the saddle joint with the internal ligament, the control pins and gears, the inner tendons, and the fully assembled acrylic bases. Tendons were adjusted manually to achieve the correct tension, ensuring that the fingers remained extended but stable.

The final hand has the following dimensions: **length**: 290 mm; **width**: 240 mm; **depth**: 60 mm (when fully extended); **weight**: 310 g.

### 4.1 Movement assessment

**FIGURE 6:**
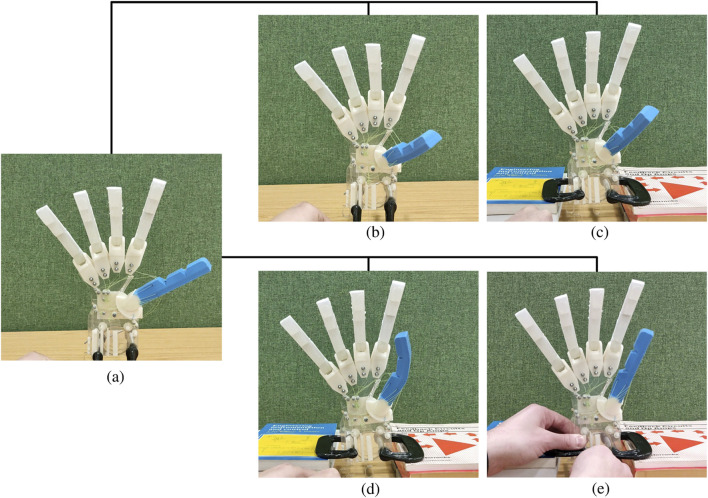
Sample of movements for the Anthro-Thumb. **(a)** Thumb in rest position. **(b)** Thumb opposition. **(c)** Thumb flexion. **(d)** Thumb gripping. **(e)** Thumb adduction.

Figure 6 demonstrates the resulting movements of the thumb after fully actuating each individual muscle equivalent, showing the rest position of the abducted thumb (a), complete thumb opposition (b), thumb flexion (c), thumb gripping (d), and thumb adduction. These individual movements can be combined to produce specific behaviors.

Table 3 compares the target values set in Section 2.2 against the results achieved during prototyping and CAD assessment. The prototype results were obtained by mounting the base of the prototype joint and fully extending the joint in each ROM direction, measuring the maximum rotation in each plane. Due to the elastic ligament, the ROM values differ from the design targets.

**TABLE 3:** ROM results for the design and testing of the Anthro-Thumb CMC joint.

Test	Adduction/abduction (deg)	Flexion/extension (deg)
Robotic thumb guidance CMC ROM (Chalon et al., 2010) (requirement)	35°/25°	15°/30°
CAD assessment	35°/25°	30°/13°
Prototype joint	40°/31°	30°/15°

The final Anthro-Thumb design allows for increased adduction, abduction, and flexion ROM compared to the target values but exhibits a reduced extension ROM.

### 4.2 Dexterity assessment

The Kapandji test was used to evaluate the Anthro-Thumb’s ability to achieve opposition and dexterity comparable to a human thumb. Specific measurements for phalanx lengths and joint ranges of the index, middle, ring, and little fingers were not taken during this experiment. Instead, the test results were recorded based solely on whether the Anthro-Thumb could achieve the required positions. This approach allowed us to focus on the functional performance of the thumb during opposition and grasping tasks, as outlined in the GRASP taxonomy. The Kapandji test is a clinical method utilized during physiotherapy to evaluate thumb rehabilitation. It is a qualitative assessment of the thumb’s ROM based on positions on the hand rather than measuring joint angles (Kapandji, 1986). This test provides a measure of the thumb’s ability to oppose the fingers.

The test assigns scores to specific locations on the hand. The subject is asked to sequentially touch these locations with their thumb. Progressing through the sequence increases the score from 1 (for the first location) to 10 (for the final location) (Kapandji, 1986). If the subject is unable to reach a specific location, the test ends and the final score is recorded (Kapandji, 1986).

**FIGURE 7:**
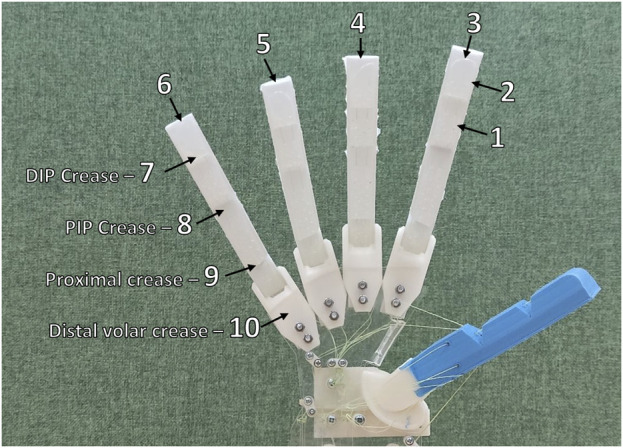
Target locations for thumb contact in the Kapandji test.

Figure 7 shows the locations on the hand identified as targets for the Kapandji test. The test begins with the thumb in a central resting position, with a human operator using the control pins to attempt to reach each of the highlighted locations in sequence without returning to the central resting position. If a location cannot be reached, the thumb is reset to the central resting position, and the test ends.

**FIGURE 8:**
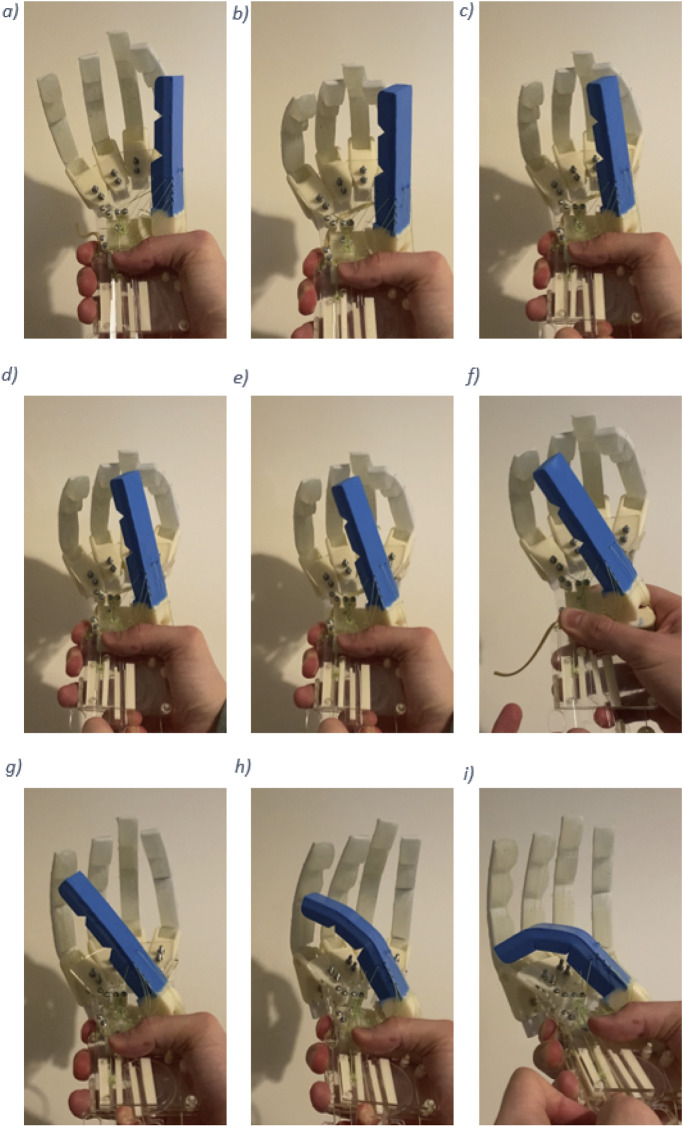
Kapandji test results. **(a)** Thumb contact with location 1. **(b)** Thumb contact with location 2. **(c)** Thumb contact with location 3. **(d)** Thumb contact with location 4. **(e)** Thumb contact with location 5. **(f)** Thumb contact with location 6. **(g)** Thumb contact with location 7. **(h)** Thumb contact with location 8. **(i)** Thumb contact with location 9.

Figures 8a–i show the successful locations reached during the Kapandji test. The hand was unable to achieve the final position in the test, as the thumb could not reach location 10 (the distal volar crease).

The hand achieved a score of 9 in the Kapandji test, successfully reaching all locations from the side of the second middle phalanx to the proximal crease of the little finger. A score of 10 was unachievable because the thumb could not contact the distal volar crease. It is believed that this limitation is due to the overall thumb design rather than the functionality of the CMC joint. Nonetheless, the achieved score of 9 demonstrates that the proposed anthropomorphic thumb design provides a high level of opposition to the four front fingers of the hand.

Table 1 compares the self-reported Kapandji test scores of popular high-end robotic hands. Although the Anthro-Thumb is a simple and cost-effective robotic thumb, its results are comparable to those of more expensive, high-end robotic hands, highlighting the effectiveness of the biomimetic design approach.

### 4.3 Force assessment

Actuation force was measured using a spring balance device. The required force to fully actuate each individual control was measured, and the maximum force required was recorded. Results are summarized in Table 4. Strength limits were not evaluated to avoid potential damage to the hand components.

**TABLE 4:** Force required for complete contraction of each thumb muscle and the front finger group. For abduction/adduction, the cable force was measured directly, excluding the mechanical effects of the gear.

Gripping	Flexion	Opposition	Abduction/adduction	Front finger group
24 N	9 N	11 N	6 N	54 N

### 4.4 Grasp assessment

Two separate grasp tests were carried out to determine the base-level performance of the hand with the *Anthro-Thumb*. A human operator was used in place of a dexterous arm. The operator used the actuator pins to move the thumb and fingers into position to grasp and lift objects off a flat surface. As noted at the beginning of Section 3.3, due to the frail components of the hand, the test was limited to a small sample of lightweight objects under 150 g.

The first round of tests was performed with lightweight 3D-printed cubes made of PLA. This provided an initial benchmark, allowing the operator to understand the controls and ensure repeatable results without (a) damaging the hand or (b) compromising results by using unorthodox grasping and lifting methods.

After completing the first set of grasping experiments, a selection of three everyday objects was tested to further evaluate the hand’s capabilities. These objects included a box of paracetamol, a roll of duct tape, and a box of dried instant oats. All items were successfully grasped and lifted from the table without compromising the structure of the hand. Dimensions for each object used in both experiments are listed in Table 5.

**TABLE 5:** Dimensions and mass of the test objects.

Object	Dimensions (H × W × D) (mm)	Mass (g)
Cube 1	30 × 30 × 30	10
Cube 2	40 × 40 × 40	20
Cube 3	50 × 50 × 50	37
Paracetamol box	68.5 × 62.5 × 20	18
Box of oats	86 × 88 × N/A	68
Duct tape	47 × 93 × N/A	114

**FIGURE 9:**
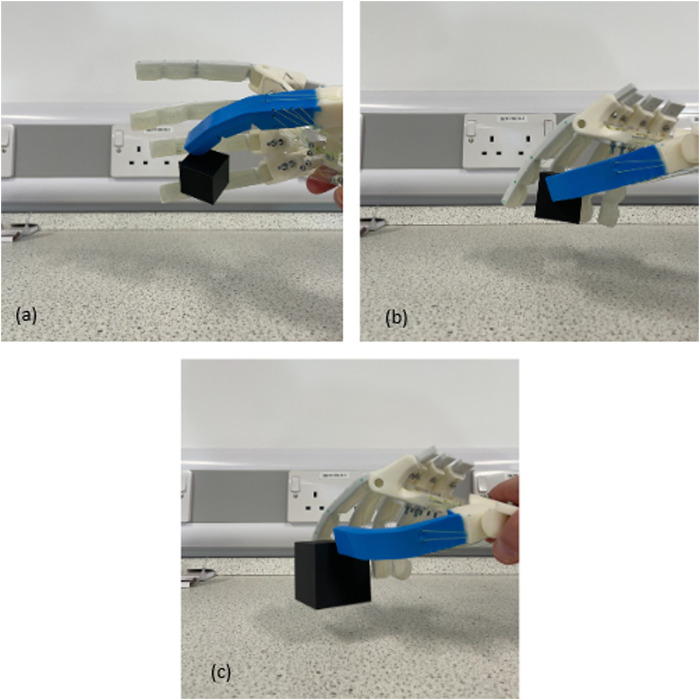
First grasping test results: **(a)** grasping a 30 × 30 × 30 cube, **(b)** grasping a 40 × 40 × 40 cube, and **(c)** grasping a 50 × 50 × 50 cube.

**FIGURE 10:**
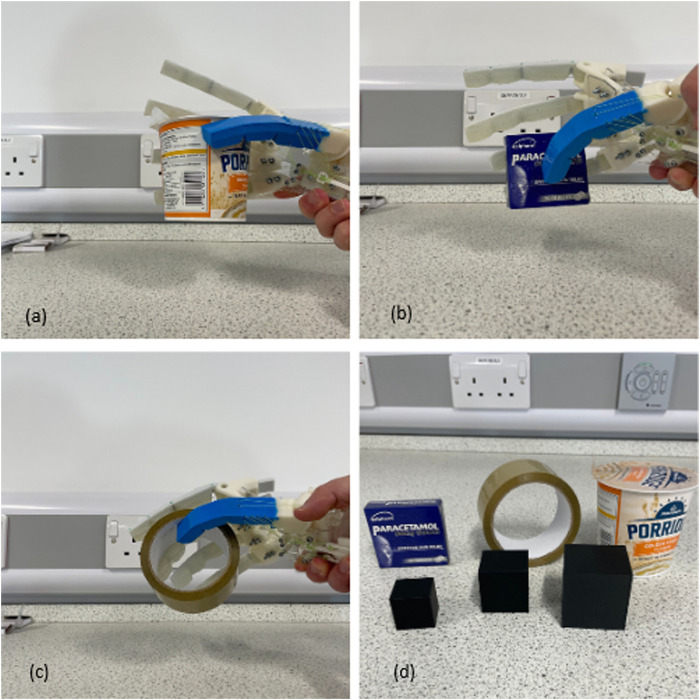
Second grasping test results: **(a)** (top left) grasping a box of oats, **(b)** (top right) grasping a paracetamol box, **(c)** grasping a roll of duct tape, and **(d)** all objects tested.

Figures 9, 10 showcase the results of the grasp tests. Some holds required skillful maneuvering by the operator. Clockwise from the figures, several common grasp techniques from the GRASP taxonomy (Feix et al., 2016) can be identified: the prismatic 3-finger grip, large diameter grip, and light tool grip.

The sphere grip and prismatic grips achieved by the Anthro-Thumb were not accomplished by the hand design proposed by Sardinha et al. (2022), highlighting the benefits of increased dexterity of the Anthro-Thumb.

Due to the frail nature of the internal control pins, extensive tests on grasping behavior could not be performed. The small size required for these pins introduces brittleness and complexity. Future iterations of this design should prioritize more robust control systems to fully explore grasping behavior.

This initial assessment demonstrated the required functionality, providing confidence in the biomimetic CMC joint concept for robotic hands. The results also identified areas for optimization, which have been incorporated into the final design.

### 4.5 Requirement assessment

Section 2.2 describes the requirements identified for the design of the Anthro-Thumb, providing a guideline for its development. A comparison of the proposed solution against its requirements is presented below.

The proposed design met all of its requirements at least at the threshold level, with two of three objectives achieved. The objective that was not achieved was related to the height of the base (trapezium carpal bone) of the CMC joint, which exceeded the 10% tolerance for the 50% adult male by +3.28 mm. However, this deviation did not appear to impact the functional performance of the developed hand.

*Requirement 1*: *size—base*. Threshold met. The joint contact surface length achieved was 25 mm (within 10% of 50% adult male), the base width was 17 mm (within 10% of 50% adult male), and the base height was 20 mm (outside 10% tolerance by +3.28 mm). The CMC joint contact areas complied with the threshold.

*Requirement 2*: *size—CMC joint*. Threshold met. The CMC joint was proportional to the rest of the hand, as confirmed by *Requirement 1* and visual inspection.

*Requirement 3*: *size—metacarpal*. Objective met. The measured thumb link length was 129 mm, the interphalangeal joint breadth was 22 mm, and the thumb circumference was 71 mm (all within 10% of 50% adult male).

*Requirement 4*: *control*. Threshold met. The muscle attachment points were accurately mapped onto the first metacarpal and palm to ensure biologically accurate force vectors.

*Requirement 5*: *biomimicry—CMC joint*. Objective met. The CMC joint achieved the required ROM to pass the Kapandji test and mimicked the functionality of a biological CMC joint.

*Requirement 6*: *connecting fixtures*. Threshold met. Connecting fixtures were validated through the successful assembly of the hand.

*Requirement 7*: *control methods*. Threshold met. The thumb was controlled by a series of pseudo-muscle cables, mimicking biological muscle forces.

## 5 Discussion

These tests demonstrated the two DOFs provided by the proposed CMC joint design. By controlling the thumb in a rotational motion through the sequence of locations in the Kapandji test, the combined use of both DOFs is evident. The Kapandji score of the Anthro-Thumb is comparable to that of more complex thumb systems, achieving similar results to the RBO Hand 2, which also failed to reach the same joint location (Deimel and Brock, 2015). Overall, the tests and assessments suggest that the proposed design provides a suitable ROM for the thumb of a soft robotic hand.

The validation of the proposed design has proved that a high level of opposition can be achieved using a biomimetic CMC saddle joint at the base of a soft robotic thumb. The use of accessible soft materials and 3D-printed components results in a relatively low-cost robotic thumb with features comparable to more advanced robotic hands.

The use of rapid prototyping techniques enabled the efficient development of a cost-effective (sub-£100) soft robotic thumb system capable of mimicking human movements. Substituting a biologically accurate capsular ligament for an internal ligament design allowed the saddle joint to retain the required ROM while preventing damage or detachment of the first metacarpal. Housing the ligament internally also reduced entanglement issues with the driving exterior ligaments, simplifying the overall design by separating the structural and actuation systems.

Achieving a score of 9 of 10 in the Kapandji test demonstrates that the thumb system can act as an opposition and enable the hand to perform multiple grasping tasks. Figures 10a, b show the proposed design’s ability to perform a pinch grasp, whereas Figure 10c displays its capability to perform a tripod grasp. This marks a significant improvement in dexterity without compromising the synergy behavior.

### 5.1 Limitations and areas for improvement

The inability to reach the final location in the Kapandji test is attributed to small-design limitations. The thumb can reach the proximal crease of the little finger, causing part of the thumb to pass directly over the distal volar crease. Increasing the mobility at the MCP joint or the IP joint could potentially allow the thumb to contact the distal volar crease.

Alternatively, inspiration can be drawn from the RBO Hand 3 (Puhlmann et al., 2022), which improved upon its predecessor (Deimel and Brock, 2015) by incorporating a rigid support structure for the soft fingers. Incorporating rigid palm opposition, as seen in the RBO Hand 3 (Puhlmann et al., 2022) or S-22 (Zhou et al., 2020), could enhance the Kapandji score and assist in grasping tasks.

These findings suggest that designs lacking actuated palms, those with under-actuation, or purely soft structures may face challenges in the Kapandji test and exhibit lower dexterity. It is worth noting that grasping forces were not exhaustively tested, and although the observed grasping positions are promising, the full functionality of the identified grasps cannot be confirmed yet.

Future work will focus on fine-tuning the MP and IP joint ROMs and conducting more rigorous testing of loading capacity to further validate the Anthro-Thumb’s performance in real-world applications. These improvements will enhance the thumb’s ability to perform a wide range of grasping tasks, as demonstrated in the Kapandji test and object-grasping experiments.

Future revisions should also address the brittleness of the actuation system by using more robust materials (e.g., metal sheets instead of acrylic) or by increasing the overall scale. Refining the control system is crucial to fully explore the grasping capabilities of the hand. The lightweight and small-scale design demonstrates potential soft and safe manipulation applications, particularly in robotics and prosthetics.

### 5.2 Control challenges and proposed solutions

During testing, it was observed that the absence of automatic tensioning for the pseudo-muscle cables caused slight looseness in the abductor pollicis brevis and adductor pollicis cables after thumb flexion. The cable representing the pseudo-flexor pollicis brevis enables flexion of the thumb toward the small finger. This looseness reduced the precision of thumb abduction and adduction control. The pseudo-abductor pollicis brevis is routed through a cable gate in the position of its biological origin (radial edge of the trapezium) before being attached to its respective gear. Although the focus of this project was on developing an anthropomorphically accurate thumb rather than its control system, refining the control mechanism is essential for further development.

To address this, several potential solutions and alternative designs are proposed:

1. **Automatic tensioning system:** incorporating a spring-loaded tensioning mechanism at the cable endpoints could maintain consistent tension during and after thumb movement.
2. **Elastic tendon elements:** using slightly elastic tendon materials in place of rigid cables could absorb minor slack caused by thumb flexion while maintaining the required tension for precise control.
3. **Differential pulley system:** introducing a differential pulley system could distribute tension dynamically across the cables such that looseness is minimized during operation.
4. **Pre-tensioning mechanism:** adding a pre-tensioning system with adjustable knobs could allow manual adjustment of cable tension before operation, offering a simple and cost-effective solution for maintaining cable tautness.

Mechanically coupling the actuation points of the thumb or combining the proposed solutions above could ensure that the thumb remains taut while adhering to the soft synergy requirements (Santina et al., 2018). However, these adjustments may introduce significant complexity to the system.

The proposed design (*Anthro-Thumb*) serves as a demonstrator for the functionality of using saddle joints at the base of soft robotic thumbs. The developed system provides the full range of thumb movement, with a total of four DOFs (two at the base and one each at the MP and IP joints), closely mirroring the behavior of a biological thumb. This work highlights the feasibility and potential of a biomimetic approach to robotic thumb design, offering a promising foundation for future developments.

## 6 Conclusion

This paper presented the concept, development, and assessment of a novel biomimetic soft robotic thumb designed to enhance a tendon-driven robotic hand. The optimization of the design was guided by initial simulation and testing findings. Through this process, a four-DOF soft robotic thumb prototype was developed, achieving a high level of opposition to the fingers of the robotic hand without the need for electronic components. The effectiveness of the novel joint system was validated through the Kapandji testing technique, providing confidence in its functionality. The grasp tests were qualitative in nature, serving as preliminary assessments of the hand’s functionality. Future work will include quantitative experiments to measure the grip strength and dexterity more precisely.

A comparative analysis with existing robotic thumb designs highlighted the advancements achieved, particularly in terms of ROM and cost-effectiveness. Additionally, the discussion on long-term durability and maintenance requirements of the soft robotic materials and components suggests that the proposed design is a viable solution for soft robotic applications.

Future work should aim to address the following:

• Increasing the loading capacity of the Anthro-Thumb.

• Introducing tensioning mechanisms for the pseudo-muscles to maintain cable precision and alignment.

• Fine-tuning the MP and IP thumb joints to ensure that the required ROM is achieved.

• Protecting the tendon cables and optimizing the internal ligament material to improve durability.

• Enhancing the control system by incorporating closed-loop control and embedding sensing systems into the silicone fingers and thumb.

• Exploring tactile sensing approaches, such as piezoelectric and capacitive sensors, to enable environmental interaction detection and achieve programmable control.

These improvements will enhance the capabilities and robustness of the biomimetic soft robotic thumb, advancing its potential applications in soft robotics and prosthetics. The proposed design lays a solid foundation for further research and development in creating cost-effective, highly functional, and adaptable robotic systems inspired by human anatomy.

## Data Availability

The original contributions presented in the study are included in the article/supplementary material, further inquiries can be directed to the corresponding author.
